# Genomic sequence of '*Candidatus* Liberibacter solanacearum' haplotype C and its comparison with haplotype A and B genomes

**DOI:** 10.1371/journal.pone.0171531

**Published:** 2017-02-03

**Authors:** Jinhui Wang, Minna Haapalainen, Thomas Schott, Sarah M. Thompson, Grant R. Smith, Anne I. Nissinen, Minna Pirhonen

**Affiliations:** 1 Department of Agricultural Sciences, FI-00014 University of Helsinki, Helsinki, Finland; 2 Herne Genomik, Neustadt, Germany; 3 The New Zealand Institute for Plant & Food Research Limited, Lincoln, New Zealand; 4 Plant Biosecurity Cooperative Research Centre, Canberra, ACT, Australia; 5 Better Border Biosecurity, Lincoln, New Zealand; 6 Management and Production of Renewable Resources, Natural Resources Institute Finland (Luke), Jokioinen, Finland; Academia Sinica, TAIWAN

## Abstract

Haplotypes A and B of ‘*Candidatus* Liberibacter solanacearum’ (CLso) are associated with diseases of solanaceous plants, especially Zebra chip disease of potato, and haplotypes C, D and E are associated with symptoms on apiaceous plants. To date, one complete genome of haplotype B and two high quality draft genomes of haplotype A have been obtained for these unculturable bacteria using metagenomics from the psyllid vector *Bactericera cockerelli*. Here, we present the first genomic sequences obtained for the carrot-associated CLso. These two genomic sequences of haplotype C, FIN114 (1.24 Mbp) and FIN111 (1.20 Mbp), were obtained from carrot psyllids (*Trioza apicalis*) harboring CLso. Genomic comparisons between the haplotypes A, B and C revealed that the genome organization differs between these haplotypes, due to large inversions and other recombinations. Comparison of protein-coding genes indicated that the core genome of CLso consists of 885 ortholog groups, with the pan-genome consisting of 1327 ortholog groups. Twenty-seven ortholog groups are unique to CLso haplotype C, whilst 11 ortholog groups shared by the haplotypes A and B, are not found in the haplotype C. Some of these ortholog groups that are not part of the core genome may encode functions related to interactions with the different host plant and psyllid species.

## Introduction

‘*Candidatus* Liberibacter solanacearum’ (CLso) was first described in connection with diseases of solanaceous crops, including potato, tomato and capsicum in New Zealand and North America [1–7]. Later, the same bacterial species was found in Europe, associated with diseases in the Apiaceae family plants carrot and celery [8–12]. Phylogenetic analysis using the combination of the 16S rRNA, 16S-23S rRNA intergenic spacer region (ISR) and 50S ribosomal protein gene sequences, revealed that the CLso bacteria found in different geographic regions were diverse and could be assigned to five separate clades: haplotype A, B, C, D or E [12,13]. CLso haplotypes A and B are associated with Zebra chip (ZC) disease of potatoes and psyllid yellows of tomato and capsicum [4], and these two haplotypes are transmitted by the tomato/potato psyllid *Bactericera cockerelli* Šulc (Hemiptera: Triozidae) in a circulative-persistent mode [2,7,14]. CLso haplotype C is associated with carrot yellowing disease in Northern Europe, where it is transmitted by the carrot psyllid, *Trioza apicalis* Förster [11,15,16]. In addition to findings in Finland, CLso haplotype C has been detected in Sweden, Norway and Germany [17–19]. CLso haplotypes D and E were first described in carrot and celery in Spain, and the psyllid *Bactericera trigonica* Hodkinson is suspected to act as a vector for these haplotypes in Spain [9,12,20]. Haplotypes D and E have also been found in carrot in France and Morocco [21–23].

In 2011, the complete genome sequence of CLso haplotype B (ZC1) was obtained via metagenomics, using DNA that had been isolated from CLso bacteria collected by immuno-capture from a pooled sample of field-captured tomato/potato psyllids and then amplified by whole genome amplification [24]. CLso ZC1 is the first and only completely assembled CLso genome to date. In 2015, two high quality draft genomes of CLso haplotype A were published. The first assembly, NZ1, was obtained using metagenomics from DNA isolated from one tomato/potato psyllid individual from a greenhouse-reared colony in New Zealand. The DNA for Illumina sequencing was amplified using whole genome amplification. The other CLso haplotype A assembly, HenneA, was obtained from DNA isolated from two tomato/potato psyllid individuals from a greenhouse-reared colony in the United States. The DNA from two psyllid individuals with high CLso titres were combined into one DNA sample for sequencing [25]. Analysis of these complete or high quality draft genomes provided insights into the biology of CLso, and revealed genetic variation between haplotypes A and B. In addition, two draft genome sequences of CLso, R1 and RSTM, were obtained from a tomato plant and a tomato/potato psyllid respectively, in California [26,27]. These two draft genomes are still highly fragmented, R1 consists of 99 contigs and RSTM consists of 26 contigs, which limits their use in genome structure analysis. However, these draft genomes provide information of the genetic variation within CLso. As genome sequences from the other haplotypes (C, D and E) were missing, a more robust analysis could not be undertaken.

Of the five haplotypes of CLso, haplotype C has the most distinct vector, the carrot psyllid *Trioza apicalis*, which occurs in the temperate and subarctic climate areas in Northern Europe, whereas the identified or suggested vectors of the other haplotypes of CLso belong to genus *Bactericera* and occur in areas with temperate or tropical climates. To determine if CLso haplotype C is genetically different from haplotypes A and B, we sequenced and assembled the genome of haplotype C and undertook genome comparisons. Two draft genome sequences of CLso haplotype C were obtained from DNA isolated from two carrot psyllid individuals from south-west Finland. One of these haplotype C draft genome sequences, FIN114, was compared with the complete genome sequence of haplotype B (ZC1) and one high quality draft genome sequence of haplotype A (NZ1). Genomic comparisons of these three haplotypes of the same bacterial species revealed the size of both the core and pan genomes, and identified potential haplotype-specific genes that may be involved in the different host plant or psyllid interactions.

## Materials and methods

### Psyllid and plant samples

All carrot psyllids were captured from the same population in a carrot field in Forssa, south-west Finland in the summer of 2012, with the permission of the land owner. Thereafter, the psyllids were reared on carrot plants (cv. Fontana) in a greenhouse in Jokioinen, Finland. In the transmission experiment, each psyllid individual was released on one carrot seedling enclosed in an insect cage [16]. After three days' exposure, psyllids were removed from the carrot plants, the DNA was extracted from the psyllids using DNeasy Blood and Tissue kit (Qiagen) according to the manufacturer's protocol, and the DNA was then eluted in 30 µl of nuclease-free water. Each DNA sample was further diluted 1/100, and 5 µl per reaction was used for quantitative PCR [16]. DNA samples from two carrot psyllid females 111 and 114 that contained a very high titre of CLso, Ct values 19.25 and 18.93 at sample dilution 10^−2^ respectively, were used as the material for sequencing (S1 Table). DNA was also extracted from the carrot plants nine weeks after the exposure to psyllids, using the CTAB method as previously described [16]. DNA from the CLso-infected carrots A2F2 and A5F2 exposed to feeding by psyllids 111 and 114, respectively, was used as a PCR template for CLso sequence validation and gap closure. DNA from a healthy control carrot A7C1 grown in an insect proof cage in a greenhouse was used as a CLso-negative PCR template.

### Genome sequencing

Whole genome amplification was conducted on the DNA of both the carrot psyllid samples 114 and 111 using a RepliG kit (Qiagen). Library construction and Illumina HiSeq2000 sequencing was conducted by Macrogen Inc. (South Korea). A 432 bp paired-end library was sequenced for sample 114 and a 670 bp paired-end library and a 3 kb mate-pair library were sequenced for sample 111. Part of the DNA sample 114 was also sequenced on two PacBio RS SMRT cells at Expression Analysis Ltd (USA) (S1 Table).

### Genome assembly and gap closure

Paired Illumina reads were adaptor-clipped using Mira v4.0.2 [28] and quality clipped using Sickle v1.33 (github.com/najoshi/sickle). Remaining intact read pairs were assembled using idba_ud v1.1.1 [29]. Contigs shorter than 200 nt were discarded. Remaining contigs with a blastn-hit against any published Liberibacter genome among the top 10 best hits, as well as any Liberibacter and related phage sequences available from RefSeq (as at May 2015) were used to extract potential Liberibacter read pairs using Mirabait from the Mira package with default settings. Resulting read pairs were assembled using SPAdes v3.6.1 [30] in MDA mode with k = 27,45,65. Contigs longer than 1kb were edited using Gap5 from the Staden package [31]. The CLso assembly of psyllid 111 DNA was used for predicting the contig joins of the other CLso assembly 114, and the contigs were ordered using Mauve v2.4.0 [32]. The draft genome sequence FIN114 was intensively re-sequenced to obtain high quality sequence that could be used in comparative genomic analyses. Primers binding to the contig ends were designed using Primer3 [33], and these primer pairs (S2 Table) were used to bridge potential gaps between the adjacent contigs of assembly FIN114 by either conventional or long-range PCR using DNA template from the psyllid sample 114. All conventional PCR and long-range PCR amplifications were performed using Phusion High-Fidelity DNA Polymerase (Thermo Scientific) according to the PCR protocol provided by the manufacturer. The PacBio reads were also used to predict joins between the pre-assembled contigs of assembly 114, and those predictions were confirmed by long-range PCR. In addition, to confirm the locations and sequences of the rRNA operons encoding for the 16S, 23S and 5S rRNAs and to determine SNPs or indels in these operons, each of three 5.6 kb gene regions was cloned in pCR-Blunt vector and re-sequenced. Primers (S2 Table) were selected to target the flanking regions beyond each rRNA operon end. All the amplified PCR products were gel-purified and extracted using QIAquick Gel Extraction Kit (Qiagen). The purified PCR products were ligated into pCR-Blunt plasmid vector included in the Zero Blunt PCR Cloning Kit (Thermo Scientific). Ten transformed *E*. *coli* Top10 (Thermo Scientific) colonies were picked for each rRNA copy. The re-constructed plasmids of each clone were isolated using QIAprep Spin Miniprep Kit (Qiagen) and analyzed by restriction enzyme digestion using FastDigest EcoRI (Thermo Scientific). The complete sequences of the inserted DNA fragments were obtained by primer walking and Sanger sequencing at Macrogen Europe (The Netherlands). To confirm the presence and locations of putative prophage regions, the joining regions between prophage and bacterial chromosomal sequences were amplified with designed primers (S2 Table) by long-range PCR similarly as described above, and the PCR products were sequenced through primer walking at Macrogen Europe. The quality clipped reads of dataset 111 were mapped against the draft genome sequence of FIN114 using BWA v0.7.12 [34]. The consensus sequence for regions with reads contiguously mapping were extracted. Regions where paired end reads indicated that contigs were joined but differences in the genomes prevented mapping were fixed manually.

### Genome annotation

Two draft genome sequences of CLso haplotype C, FIN114 and FIN111, were annotated using the NCBI Prokaryotic Genome Annotation Pipeline (NCBI_PGAP) and deposited in the GenBank under accession numbers LWEB00000000 and LVWB01000000, respectively.

### Phylogenetic analysis

To construct a phylogenic tree, four species of ‘*Candidatus* Liberibacter’ and the species *Liberibacter crescens* were compared with each other and to twelve closely related Alphaproteobacteria, *Agrobacterium tumefaciens*, *Sinorhizobium meliloti*, *Brucella melitensis*, *Brucella abortus*, *Bartonella quintana*, *Bartonella bacilliformi*, *Bartonella henselae*, *Bartonella vinsonii*, *Mesorhizobium loti*, *Mesorhizobium opportunistum*, and two *Phyllobacterium* species. *Rhodospirillum rubrum* from the order Rhodospirillales of the class Alphaproteobacteria was used as the outgroup (S3 Table). Protein coding genes were clustered into ortholog groups using OrthoMCL v2.0.9 [35]. In total, the annotations of 96 single copy ortholog groups from all the bacterial genomes included in the analysis were manually checked. Any ortholog group related to an unknown function or possible horizontal gene transfer was removed from the set before analysis. Finally, 88 ortholog groups were aligned using Muscle v3.8.31 [36], and then the multiple alignments were trimmed and concatenated into a supermatrix. The best amino acid substitute model for this supermatrix was determined using ProtTest v3.4.1 [37]. Maximum-likelihood tree was constructed using RAxML v8.2.0 [38] and applying ‘PROTGAMMAIWAGF’ setting.

### Genome comparisons

As intensive sequence validations and gap closure had been conducted on assembly FIN114, this draft genome sequence was used for genome comparisons. The predicted protein sequences of FIN114 were analyzed using the Kyoto Encyclopedia of Genes and Genomes (KEGG) pathway maps to reconstruct the metabolic pathways [39] of CLso haplotype C. The result was compared to the pathways predicted by KEGG for the curated complete ‘Liberibacter’ genomes, including ‘*Ca*. Liberibacter solanacearum’ (ZC1), ‘*Ca*. Liberibacter asiaticus’ (psy62, Gxpsy, Ishi-1), ‘*Ca*. Liberibacter americanus’ (Sao Paulo), ‘*Ca*. Liberibacter africanus’ (PTSAPSY) and *Liberibacter crescens* (BT-1). Closer comparisons of the mevalonate pathway between ‘Liberibacter’ and *Marinobacterium* species was performed through the EcoCyc database [40]. To obtain an overall view of the genome synteny between the three CLso haplotypes, the sequence FIN114 was aligned against the haplotype B ZC1 genome (accession GCA_000183665.1) and the haplotype A NZ1 genome (accession GCA_000968085.1) using progressive Mauve algorithm from Mauve v2.4.0 [32]. Because the genome assemblies of FIN114 and NZ1 are in contigs, the complete genome sequence ZC1 was set as the reference. Long-range PCR was used to confirm the re-arrangement of several genomic regions in FIN114 in relation to ZC1. The local conserved blocks (LCBs) and the guide tree were determined by the progressive Mauve algorithm and visualized using the R package genoPlotR v0.8.4. The annotated protein sequences of assemblies NZ1, ZC1 and FIN114, representing CLso haplotypes A, B and C, respectively, were clustered into ortholog groups using OrthoMCL. Short open reading frames with less than 50 amino acid codons were filtered away. In the subsequent all-blast-all step using Blastp, the required minimum coverage over both query and subject sequence was set at 70%. The ortholog clustering result of OrthoMCL analysis was converted into an orthologs vs. haplotype binary (1/0: present/not present) matrix, and visualized as a Venn diagram using the R package eVenn v2.3.2. Those genes identified as singletons (i.e. genes not assigned to any ortholog group) in each genomic assembly were further filtered by Blastn against the other two CLso genome sequences, and any Blastn-hit with e-value lower than 1e^-11^ and coverage more than 50% over query was discarded as being a potential homolog. These remaining singletons were then screened by Blastn against NCBI nr database using the same threshold to identify those singletons that show similarity to sequences of other species, and the remaining singletons were considered as FIN114 specific. The same Blastn filtering was applied to the ortholog groups that were present in NZ1 and ZC1, but not in FIN114. Since the integrity of assembly FIN111 was not confirmed by PCR, it was not used in Mauve alignments, but the putative haplotype specific genes or ortholog groups identified by comparisons between FIN114, NZ1 and ZC1 were also compared by reciprocal Blastp (identity above 40% and 70% coverage on both query and subject) against the FIN111 protein dataset. For these haplotype specific genes or ortholog groups, we used Blast2GO [41] to annotate gene functions via a workflow of BLAST, Gene Ontology (GO) mapping, and InterProScan. SignalP v4.1 [42] and SecretomeP v2.0 [43] were used to identify a signal peptide or a non-classical protein secretion signal respectively, as previously described [44]. For the putative protein AYJ09_01490 and its homologs, amino acid sequence alignment was performed using Clustal X [45] and secondary structure prediction was performed using Jpred4 [46]. The complete prophage regions of FIN114, NZ1 [25] and ‘*Ca*. Liberibacter africanus’ PTSAPSY [47] were predicted using PHASTER [48]. The complete prophage sequences that have been characterized in previous studies, P1 and P2 from ZC1 [24], SC1 and SC2 from ‘*Ca*. Liberibacter asiaticus’ UF506 [49], FP2 from ‘*Ca*. Liberibacter asiaticus’ psy62 [50], SP2 from ‘*Ca*. Liberibacter americanus’ Sao Paulo [51], LC1 and LC2 from *Liberibacter crescens* BT-1 [52] were extracted from the genome sequences. Because the prophage genomes show mosaic architectures between the different strains, a comparison method that requires long sequence alignments between the genomes could not be used. Instead, pairwise calculation of tetra-nucleotide frequencies (TETRA) between the ‘Liberibacter’ prophage sequences was employed to estimate their relationships, as this analysis is independent of longer sequence alignments. The correlation coefficients of the TETRA of all those selected complete prophage sequences were calculated using Python package pyani (github.com/widdowquinn/pyani) and the result was visualized using R package ggplot2 v2.1.0.

## Results

### Genome features

**Fig 1:**
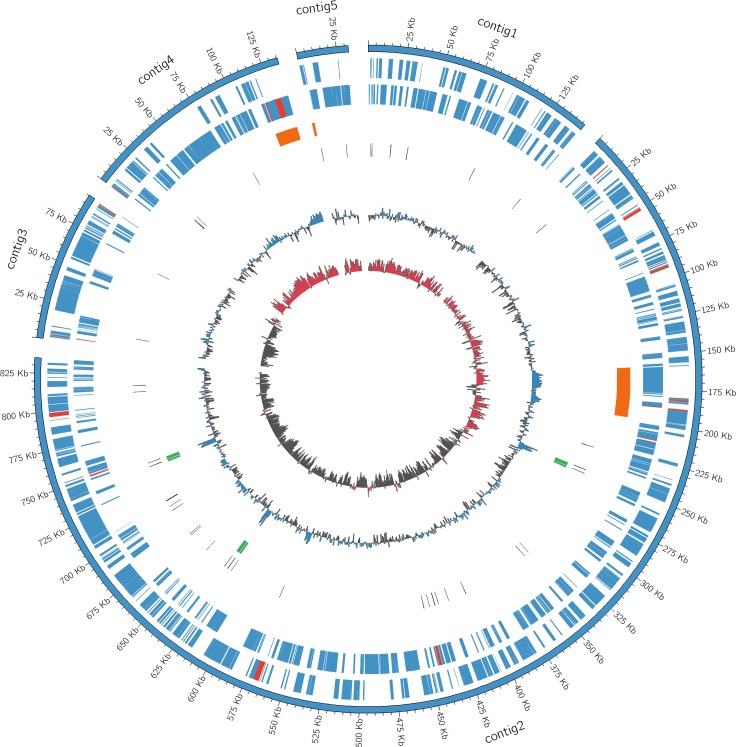
Circular representation of the draft genome sequence of ‘*Candidatus* Liberibacter solanacearum’ haplotype C FIN114. The circles represent, from outer to inner, protein coding genes on the forward strand and the reverse strand, prophage regions, tRNA, rRNA, %G+C content and GC-skew. The loci indicated with red color represent the putative haplotype C specific genes.

The assemblies FIN114 and FIN111 have 5 and 15 non-redundant contigs respectively, each with 300 times average coverage. The draft genome FIN114 has a GC content of 35.2% and a length of 1.24 Mbp, encoding 1067 predicted proteins. The draft genome FIN111 has a GC content of 34.9% and a length of 1.20 Mbp, encoding 1040 predicted proteins. The average nucleotide identity (ANI) between these two assemblies is 99.87% which shows they are highly similar. The draft genome FIN114 contains one complete prophage region, designated as phage A, in contig 2, and one partial prophage, designated as phage B, between contigs 4 and 5 (Fig 1). The prophage A is 38.3 kb long and has a GC content of 41.0%.

### CLso rRNA operons

Like the previously sequenced ‘*Candidatus* Liberibacter’ genomes, the CLso haplotype C draft genome FIN114 also contains three rRNA operons. Each of the three rRNA operons, named 16SA, 16SB and 16SC, and their variable flanking sequences were amplified by long-range PCR, and contigs with sizes 6975 bp, 6298 bp and 6825 bp assembled via primer walking and sequencing. After complete sequence alignment and removing the variable flanking sequences, the size of the rRNA operon was determined to be 5670 bp, with almost identical sequence between the three copies. They all include the genes 16S rRNA, tRNA-Ile, tRNA-Ala, 23S rRNA, 5S rRNA and tRNA-Met in the same order. Only one polymorphic site was found within the 23S rRNA sequence where 16SA differs from 16SB and 16SC at position 3848: C/T. The sequences of the three rRNA operons of haplotype C were deposited at GenBank under accession numbers KX431889, KX431890 and KX431891.

### Phylogenetic tree

**Fig 2:**
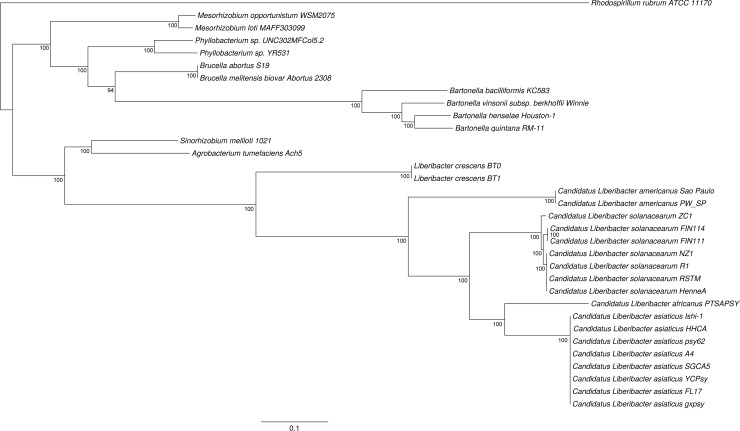
Phylogenetic tree constructed of 88 protein-coding genes from 33 bacterial genome datasets, belonging to genera ‘*Candidatus* Liberibacter’, *Liberibacter*, *Agrobacterium*, *Sinorhizobium*, *Brucella*, *Bartonella*, *Mesorhizobium*, *Phyllobacterium* and *Rhodospirillum*. The 88 ortholog groups were aligned using Muscle v3.8.31, and these multiple alignments were trimmed and concatenated into a supermatrix. The best amino acid substitute model was determined using ProtTest v3.4.1. Maximum-likelihood tree was constructed using RAxML v8.2.0 with ‘PROTGAMMAIWAGF’ setting. The numbers shown next to the branches indicate the percentage of bootstrap support values (1000 replicates). The branch lengths indicate the evolutionary distance as the number of base substitutions per site.

A phylogenic tree for the genus ‘*Candidatus* Liberibacter’ and related species was constructed using the supermatrix approach and based on 88 single-copy ortholog groups (Fig 2). The tree obtained is robust, with strong bootstrap support. The analysis clearly shows that the ‘Liberibacter’ species are divided into two sub-clades. All the CLso clades and the huanglongbing-associated species ‘*Candidatus* Liberibacter africanus’ (CLaf), ‘*Candidatus* Liberibacter asiaticus’ (CLas) and ‘*Candidatus* Liberibacter americanus’ (CLam) clustered into the plant pathogen sub-clade of ‘*Candidatus* Liberibacter’. *Liberibacter crescens*, which is non-pathogenic and culturable, was the only member of the other sub-clade. As expected, CLso haplotype C sequences FIN114 and FIN111 and all the other CLso haplotypes together form the CLso clade, in which the three haplotypes further differentiate into three distinct haplotype clades with strong bootstrap support.

### Prophage sequence

**Fig 3:**
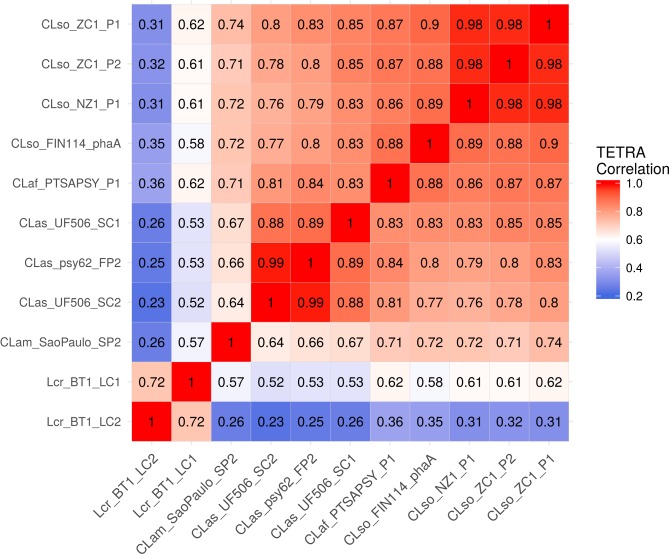
Tetranucleotide frequency correlation coefficients (TETRA) of eleven prophage sequences from ‘*Candidatus* Liberibacter’ species and *Liberibacter crescens*. CLso, ‘*Candidatus* Liberibacter solanacearum’; CLaf, ‘*Candidatus* Liberibacter africanus’; CLas, ‘*Candidatus* Liberibacter asiaticus’; CLam, ‘*Candidatus* Liberibacter americanus’; and Lcr, *Liberibacter crescens*.

The TETRA correlation coefficient values of 11 complete prophage sequences (Fig 3) show that CLso FIN114 prophage A sequence is highly similar to the prophage sequences from NZ1 (P1) and ZC1 (P1 and P2) with correlation coefficient values between 0.88 to 0.90, and is less correlated to prophage sequences SC1, SC2 and PF2 from CLas with values between 0.77 to 0.83. The prophage sequence of CLaf PTSAPSY is correlated to all CLso prophage sequences with correlation coefficient values from 0.86 to 0.88, and less correlated to CLas prophages. This was unexpected, since the core genome of CLaf is more closely related to CLas than to CLso (Fig 2). All the prophages found in the ‘*Ca*. Liberibacter’ species and *L*. *crescens* belong to order Caudovirales family Podoviridae.

### Differences in genome organization between CLso haplotypes A, B and C

**Fig 4:**
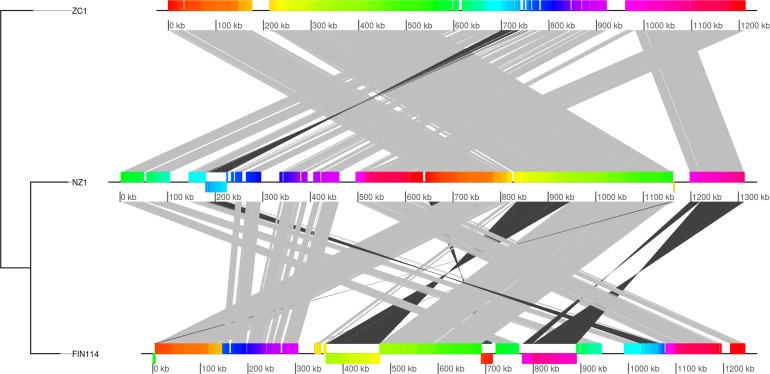
Multiple genome alignment of the ‘*Candidatus* Liberibacter solanacearum’ haplotypes A, B and C. Comparison was made between the complete genome sequence of CLso haplotype B ZC1 (top), and the draft genome sequences of haplotype A NZ1 (middle) and haplotype C FIN114 (bottom). Lines connect the homologous local conserved blocks (LCBs) between the genomes, with grey color showing connection between LCBs that are in the same orientation and black color showing connection between LCBs that are in the opposite orientation. The tree shown on the left represents the guide tree of the progressive Mauve alignment.

The average nucleotide identity (ANI) is 97.70% between ZC1 and FIN114, and 97.91% between NZ1 and FIN114, which indicates that these lineages belong to the same species. The ANI result agrees with the multi-locus phylogeny tree (Fig 2) showing that haplotype C clade is more closely related to the clade of haplotype A, and agrees with the guide tree of Mauve alignment (Fig 4), which also suggests that NZ1 and FIN114 are more closely related to each other than to ZC1. In general, the Mauve alignment shows synteny between ZC1 and NZ1, except one major rearrangement in the contig 2 of NZ1, which was split into contig 2a and 2b for the Mauve alignment in the previous report [25], and one inverted local conserved block (LCB) in contig 1 of NZ1. In contrast, the Mauve alignment analysis between ZC1 and FIN114 reveals multiple large genome rearrangements (Fig 4). Most of these large rearrangements are located within contig 2, which is the largest contig (834 kbp) of the FIN114 genome sequence. There are multiple large genomic regions within FIN114 contig 2 that are in the reverse orientation, and in different relative positions, to that in ZC1. One large inverted region, starting approximately at nucleotide position 364000, is next to prophage A, and is thus likely to be the result of a phage-mediated rearrangement. For another large inverted region, starting at position 690600, the homologous region in ZC1 has stretches of repetitive sequence on both sides. These differences in the genomic organization in FIN114 in comparison with ZC1 were all confirmed by long-range PCR and Sanger sequencing. The largest inversion, located approximately between positions 770000 and 888000 in FIN114, is between two identical rRNA operons, 16SB and 16SC, and thus the inversion is probably a result of a recombination event between these two rRNA operons. The variable flanking sequences of these two rRNA operons were confirmed by sequencing of the cloned DNA fragments. The genomic region homologous to this inverted region is found in contig 5 of NZ1, and between nucleotides 980000 and 1095000 of the ZC1 genome.

### Haplotype C core genome

There are no significant differences in the core genome gene content between the haplotype B ZC1 genome and the haplotype C assembly FIN114. Thus, CLso haplotype C is likely to have a similar capacity for biosynthesis, metabolism and secretion as the haplotype B. However, new analysis of the biosynthetic and metabolic pathways revealed some differences from the previous analyses of ZC1 [24,53]. Haplotype C harbors genes to synthesize eight amino acids, aspartate, glutamate, glutamine, threonine, lysine, arginine, serine and glycine, either *de novo*, or from metabolic intermediates, like ZC1. Both ZC1 and FIN114 lack the genes required for the conversion of pyruvate to alanine by transaminase (EC 2.6.1.2) and alanine dehydrogenase (EC 1.4.1.1), suggesting that they are deficient in *de novo* synthesis of alanine from pyruvate. However, both ZC1 and FIN114 retain the genes encoding for selenocysteine lyase (EC 4.4.1.16) and cysteine desulfurase (EC 2.8.1.7), enabling them to produce alanine through deselenization of selenocysteine, or via desulfuration of cysteine. Both ZC1 and FIN114 still retain the genes for the glycolytic pathway except for the key enzyme glucose-6-phosphate isomerase (EC 5.3.1.9), which converts α-glucose-6-phosphate to β-fructose-6-phosphate. Unlike most of the bacteria that harbor genes encoding the 1-deoxy-D-xylulose-phosphate (DOXP) pathway for isoprenoid biosynthesis, FIN114 and ZC1 have a small gene cluster (AYJ09_00845-AYJ09_00870) which encodes the mevalonate pathway for isoprenoid biosynthesis. This mevalonate pathway is present in the other ‘*Candidatus* Liberibacter’ species as well, whereas it is not found in the other Rhizobiales [54]. The mevalonate pathway gene cluster of CLso shares the same conserved gene order and also over 50% amino acid identity with that of the members of Gammaproteobacteria genus *Marinobacterium*, including *M*. *jannaschii* DSM 6295 (GenBank accession JHVJ00000000), *M*. *litorale* DSM23545 (GenBank accession AUAZ00000000), and *M*. *stanieri* S30 (GenBank accession AFPL00000000).

### Differences in gene contents between the three haplotypes of CLso

**Fig 5:**
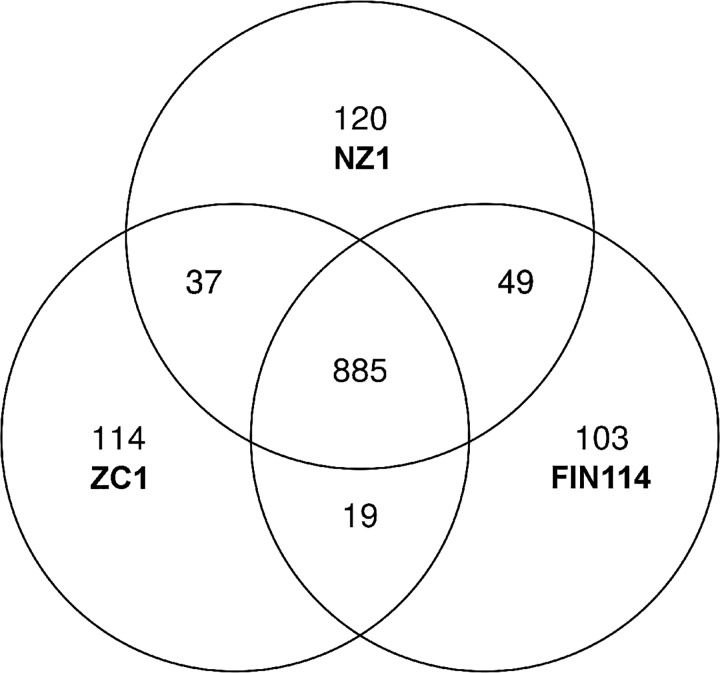
Venn diagram showing the results of OrthoMCL analysis with three ‘*Candidatus* Liberibacter solanacearum’ genomes. The figures indicate the number of ortholog groups before Blastn filtering.

The three CLso genome assemblies NZ1, ZC1 and FIN114, representing the haplotypes A, B and C, respectively, share a core-genome that consists of 885 ortholog groups, and form a CLso species pan-genome that consists of 1327 ortholog groups (Fig 5). Since the OrthoMCL analysis is performed with translated amino acid sequences, the number of ortholog groups can be different from the number of predicted genes, which also includes pseudogenes in the assembly FIN114. After Blastn filtering of the nucleotide sequences, there were 30 protein coding genes that were present in the FIN114 and absent from NZ1 and ZC1 (Table 1). Nineteen of these 30 genes were also found in the assembly FIN111 which lacks the prophage regions. Twenty-four of the 30 genes were located in contig 2, three in contig 3, and another three in contig 4 of FIN114. Two of the 30 genes (AYJ09_03220 and AYJ09_03225) were found in CLas and one (AYJ09_04845) was found in CLaf, and thus 27 genes can be considered as potential CLso haplotype C specific genes. Five of the haplotype C specific genes are putative members of restriction-modification (R-M) systems, and four of them form two complete type II R-M systems, which were not found in the CLso haplotypes A and B. Haplotype A strain NZ1 was also found to harbor two extra strain-specific R-M systems not present in the other ‘*Ca*. Liberibacter’ genomes. The other 25 genes not present in haplotypes A and B were annotated as hypothetical proteins, of which seven contain a putative secretion signal. Six of the hypothetical protein genes are located within the prophage A region and two are located within the prophage B region of FIN114. One of the genes within the prophage A of FIN114, AYJ09_01490, was found to encode a domain with a strong amino acid sequence homology (30% identity and over 70% query coverage) to the isoform 4G of the eukaryotic translation initiation factor (eIFiso4G) present in plants. The small protein encoded by FIN114 covers a part of the MIF4G domain (S1 Fig) that forms a multi-helix structure and is located on the surface of the eIFiso4G protein according to the predicted 3D structure [55]. Another hypothetical protein (AYJ09_01545) encoded in the prophage A region is a putative transferase with LbetaH domain and has over 50% identity (with an expect value of 1e^-33^) with a phage-related hypothetical protein of *Bartonella* species.

**Table 1:** Genes present in ‘*Candidatus* Liberibacter solanacearum’ haplotype C and absent from haplotypes A and B, with predicted protein products and secretion signals.

Gene Identifier	Product size (amino acids)	Annotation	Blastn hit to other Liberibacter (1e^-11^)^a^	SecretomeP2.0	SignaIP4.1 (D-cutoff 0.57)
AYJ09_00740	150	hypothetical protein	-	+	-
AYJ09_00930	77	hypothetical protein	-	+	-
AYJ09_00935	213	N6 adenine-specific DNA methyltransferase	-	-	-
AYJ09_00940	236	type-2 restriction enzyme HaeIII	-	-	-
AYJ09_01090	149	hypothetical protein	-	+	-
AYJ09_01105	361	HNH endonuclease	-	-	-
AYJ09_01490	110	hypothetical protein	-	-	-
AYJ09_01505	92	hypothetical protein	-	-	-
AYJ09_01510	68	hypothetical protein	-	-	-
AYJ09_01535	113	hypothetical protein	-	-	-
AYJ09_01540	115	hypothetical protein	-	-	-
AYJ09_01545	107	hypothetical protein	-	-	-
AYJ09_01670	73	hypothetical protein	-	-	-
AYJ09_01675	60	hypothetical protein	-	-	-
AYJ09_02650	242	type-2 restriction enzyme eco47II	-	+	-
AYJ09_02655	333	C-5 cytosine-specific DNA methyltransferase	-	-	-
AYJ09_03215	72	hypothetical protein	-	-	-
AYJ09_03220	564	hypothetical protein	+	+	-
AYJ09_03225	408	hypothetical protein	+	+	-
AYJ09_04045	186	hypothetical protein	-	-	+
AYJ09_04050	69	hypothetical protein	-	-	-
AYJ09_04230	231	hypothetical protein	-	-	-
AYJ09_04235	305	hypothetical protein	-	-	-
AYJ09_04245	162	hypothetical protein	-	-	-
AYJ09_04430	116	hypothetical protein	-	-	-
AYJ09_04435	76	hypothetical protein	-	+	-
AYJ09_04835	175	hypothetical protein	-	-	-
AYJ09_04845	88	hypothetical protein	+	-	-
AYJ09_05395	163	hypothetical protein	-	-	-
AYJ09_05425	1368	hypothetical protein	-	-	-

^a^ Other than ‘*Ca*. Liberibacter solanacearum’ haplotypes A and B.

There are 271 ortholog groups found in haplotypes A and B, either in both or in at least one, that were not present in haplotype C. After the Blastn filtering, there were twelve ortholog groups that were common to the ZC1 and NZ1 genomes and were not found in FIN114 (Table 2). However, one of these 12 ortholog groups, ortholog group 991 (DJ66_RS00890, CKC_RS03350), was found in FIN111. Eleven of the 12 ortholog groups were annotated as hypothetical proteins: the exception was ortholog group 1003 that encodes for 3-methyladenine DNA glycosylase, which is involved in base excision repair of alkylation damage in DNA. Two small 79 amino acid proteins encoded by ortholog group 986 were found to contain a recognizable N-terminal signal peptide. The ortholog group 22 (DJ66_RS00555, DJ66_RS05330, CKC_RS00980, CKC_RS05675), of which both NZ1 and ZC1 harbor two copies, encodes a hypothetical protein with weak identity (e-value 4e^-05^) to a conserved domain with unknown function (pfam04859). The proteins encoded by ortholog group 987 share over 40% amino acid sequence identity with a phage-related hypothetical protein of *Bartonella* species. The proteins encoded by ortholog group 1000 are predicted to belong to the DUF1640 superfamily (pfam07798) with two coiled-coil structures. The gene CKC_RS03570 of the ortholog group 1000 is located in a phage remnant region (779000…784000) in ZC1 and flanked by tRNA-Ser gene, repetitive sequences and two phage-related primase genes. The other gene of this ortholog group 1000 is located within the prophage P2 region in NZ1.

**Table 2:** Ortholog groups (OG) present in both ‘*Candidatus* Liberibacter solanacearum’ haplotypes A and B but not in haplotype C.

OG_ID	Gene 1	Gene 2	Gene 3	Gene 4	OG annotation	SecretomeP2.0	SignaIP4.1 (D-cutoff 0.57)
OG_22	LsoZ|CKC_RS00980	LsoZ|CKC_RS05675	LsoN|DJ66_RS00555	LsoN|DJ66_RS05330	hypothetical protein	-	-
OG_37	LsoZ|CKC_RS00945	LsoZ|CKC_RS05645	LsoN|DJ66_RS00585		hypothetical protein	-	-
OG_39	LsoZ|CKC_RS01010	LsoZ|CKC_RS05705	LsoN|DJ66_RS00525		hypothetical protein	-	-
OG_985	LsoN|DJ66_RS00550	LsoZ|CKC_RS00985			hypothetical protein	-	-
OG_986	LsoN|DJ66_RS00570	LsoZ|CKC_RS00955			hypothetical protein	-	+
OG_987	LsoN|DJ66_RS00580	LsoZ|CKC_RS00950			hypothetical protein	-	-
OG_990	LsoN|DJ66_RS00860	LsoZ|CKC_RS03375			hypothetical protein	-	-
OG_991^a^	LsoN|DJ66_RS00890	LsoZ|CKC_RS03350			hypothetical protein	-	-
OG_998	LsoN|DJ66_RS01120	LsoZ|CKC_RS03490			hypothetical protein	-	-
OG_999	LsoN|DJ66_RS01125	LsoZ|CKC_RS03495			hypothetical protein	-	-
OG_1000	LsoN|DJ66_RS01540	LsoZ|CKC_RS03570			DUF1640 domain-containing protein	-	-
OG_1003	LsoN|DJ66_RS02910	LsoZ|CKC_RS05510			3-methyladenine DNA glycosylase	-	-

^a^ OG found in FIN111.

## Discussion

In this study, two draft genome sequences of CLso haplotype C, FIN114 and FIN111, were obtained by metagenomics from individual carrot psyllids, and compared with closely related bacterial genomes. Both Illumina and PacBio sequencing were required to obtain the primary assemblies, and then gap closure of assembly FIN114 was accomplished by resequencing of approximately 10% of the genome through PCR and primer walking. Pairwise genomic comparisons by ANI suggest that CLso haplotype C FIN114 genome sequence is more closely related to haplotype A NZ1 than to haplotype B ZC1, and the same clustering was observed in the maximum likelihood phylogenetic tree based on 88 ortholog groups of the bacterial core genome. However, the TETRA correlation coefficients of the prophage regions of ‘Liberibacter’ species revealed that the prophage sequence of haplotype A NZ1 is more closely related to the prophages of haplotype B ZC1 than to the prophage A of FIN114. This suggests that the phage related regions have evolved differently from the bacterial core genome. The maximum likelihood phylogenetic tree shows that the ‘Liberibacter’ clade is clustered within the family Rhizobiaceae, which agrees with the earlier Bayesian phylogeny tree based on 94 conserved single-copy ortholog groups [56]. In contrast to this multi-locus analysis, a phylogenic analysis based on one gene, the 16S rRNA gene, placed the genus ‘*Ca*. Liberibacter’ outside of the family Rhizobiaceae [57]. Species tree—gene tree conflict may be caused by several reasons, but with closely related species the 16S rRNA gene sequence may not contain enough sequence variation and thus does not give enough information to construct a reliable phylogeny tree. It has been demonstrated that a species tree based on genome-wide datasets gives a better resolution within the class Alphaproteobacteria [58]. Also, when there are several copies of the rRNA genes within a species the copies may not be identical. The three rRNA operon sequences of FIN114 only had one polymorphic site within the 23S rRNA gene. In contrast, 47 polymorphic sites were found within the three rRNA operons in the haplotype B genome [24]. These differences between the three rRNA operons might be explained by the heterogeneous nature of the CLso DNA used for sequencing of the haplotype B genome. The haplotype B genome sequence was derived from a pooled psyllid sample that may have contained a more diverse CLso population than the single psyllid individuals used for sequencing the haplotype C FIN114, the haplotype A NZ1 genome or the two psyllid individuals combined for sequencing HenneA genome. The cloned rRNA operon sequences of haplotype C also differ from the haplotype B at a site that affects the PCR-based detection of CLso. The commonly used 16S reverse primer OI2c (5'-GCCTCGCGACTTCGCAACCCAT-3') was initially designed for detection of ‘*Ca*. Liberibacter asiaticus’ and ‘*Ca*. Liberibacter africanus’ [59,60], and it has also been widely used for detection of CLso for years. However, the primer OI2c has one nucleotide mismatch with the CLso haplotype C sequence: 5'-GCCTC(G/A)CGACTTCGCAACCCAT-3'. This mismatch impairs the specificity of PCR amplification when a plant or psyllid DNA sample contains competing bacterial DNA and the titer of CLso haplotype C is low (unpublished data). Therefore, to improve the PCR performance in CLso haplotype C detection, the primer OI2c was replaced with a specific primer Lsc2 [61].

As a result of the intracellular parasitic lifestyle of CLso, its genome has undergone reduction in both size and the gene content, which has reduced its capacity in metabolism and biosynthesis [24,62]. For example, the capacity to undertake carbohydrate metabolism is limited. In general, glucose is the main monosaccharide processed by the glycolytic pathway. However, CLso lacks the key enzyme glucose-6-phosphate isomerase (EC 5.3.1.9), suggesting it cannot convert α-glucose-6-phosphate to β-fructose-6-phosphate. However, it may bypass this step via the pentose phosphate pathway [24]. The genome of *Liberibacter crescens* contains the gene encoding for glucose-6-phosphate isomerase, whereas all the unculturable plant disease-associated ‘*Candidatus* Liberibacter’ species lack this gene [53]. Loss of this gene may be associated with a change in the bacterial lifestyle, when the bacterium became an obligate parasite. However, these bacteria have retained the capability of converting β-D-glucose to β-D-glucose-6-phosphate and generating β-D-fructose-6-phosphate via the pentose phosphate pathway, to bypass the upstream step of the glycolytic pathway. Thus, after the gene loss event these ‘*Ca*. Liberibacter’ species relied on β-D-glucose for glycolysis instead of α-D-glucose. Besides glycolysis, β-D-glucose is also a substrate for callose and cellulose biosynthesis in plants. Callose deposition and cell wall enhancement at pathogen infection sites are common responses of pattern-triggered immunity (PTI) of plants. Callose deposition has also been observed in response to CLso flg22 peptide when infiltrated into leaves of tobacco, tomato and potato plants [63]. It is possible that the ‘*Ca*. Liberibacter’ species specifically use β-D-glucose as their main source of reduced carbon for glycolysis to reduce the amount of free β-D-glucose that could serve as the substrate for PTI response-related plant cell wall fortification. CLso also has reduced capacity to synthesize amino acids, vitamins and cofactors due to gene losses in several biosynthetic pathways. In general, free-living bacteria may utilize variable carbon sources derived from central metabolism as precursors for amino acid biosynthesis. Commonly used precursors include pyruvate, oxaloacetate, 2-ketoglutarate, 3-phosphoglycerate, phosphoenolpyruvate, erythrose-4-phosphate and ribose-5-phosphate [64]. However, both the haplotype B ZC1 and haplotype C FIN114 genomes only retain the genes for utilization of oxaloacetate, 2-ketoglutarate and 3-phosphoglycerate as precursors for *de novo* synthesis of amino acids. Although ZC1 and FIN114 lack the genes for *de novo* synthesis of alanine, they harbor genes for converting selenocysteine and cysteine into alanine. The enzyme selenocysteine lyase is also found in another insect-transmitted plant phloem-inhabiting bacterium, *Candidatus* Phytoplasma asteris, which has an even smaller genome (860kbp) than CLso [65], suggesting this enzymatic activity is important for bacterial survival.

CLso species harbor a gene cluster encoding for the mevalonate pathway that may have been acquired through an ancient horizontal gene transfer. The closest homologs for these genes were found in *Marinobacterium* species that were isolated from marine environments and seawater [66–70]. Predatory bacteria belonging to Alphaproteobacteria and Deltaproteobacteria also harbor genes of this specific mevalonate pathway, which gives an advantage over the DOXP pathway by conserving energy when the substrate (aceto)acetyl-coA can be derived from the prey bacteria [71]. As obligate parasites, CLso and the other ‘*Ca*. Liberibacter’ species also share many other genetic signatures with the obligate predatory bacteria, including reduced capacity in metabolic and synthetic pathways. All of these bacteria have multiple von Willebrand factor (vWF) type A domain containing proteins (AYJ09_00680, AYJ09_02790, AYJ09_04130 in FIN114) that may facilitate cell to cell adhesion, and several proteases and peptidases that are related to the prey cell modification and degradation [71,72]. For example, CLso FIN114 possesses genes for M23 zinc metalloprotease (AYJ09_02825) and RTX toxin (AYJ09_02810). Within plants, ‘*Ca*. Liberibacter’ species are restricted to the phloem sieve cells, suggesting that they may derive nutrients directly from the sieve cell cytoplasm, while in psyllids they are likely to derive nutrients either from the psyllid cells or from the psyllid mutualist bacteria, such as *Candidatus* Carsonella ruddii [73].

The observed differences at both the genome and gene level between the haplotype C and haplotypes A and B that infect solanaceous plants may be related to the adaptation of the bacteria to different host plants and different psyllid species. Genes only present in the haplotype C were found, and among them five R-M system related genes were identified. Previously, a lineage-specific R-M system has been identified in *Staphylococcus aureus* [74]. R-M systems are common in many bacteria, and their role may not be limited to protecting the bacterial genome against phage infection. In some cases, they can also behave as mobile genetic elements and contribute to the reshaping of the bacterial genome [75]. Although the biological functions of the strain-specific R-M systems are still unclear, their presence suggests that the genomes of the strains harboring them could have methylation patterns different from the other strains. This could reduce the possibility of horizontal gene transfer between the strains in case two strains of the same species co-infect the same host organism. Of the other haplotype C-specific genes, eight genes annotated as hypothetical proteins were located within the prophage-related regions. These genes could be potential candidates for genes encoding functions required in the plant-bacteria or insect-bacteria interactions, since prophage regions have been previously found to contribute to the bacterial virulence. In ‘*Ca*. Liberibacter asiaticus’ UF506 the prophage SC2 carries a small gene SC2_gp095 that was identified to encode a peroxidase enzyme. Transgenic expression studies revealed that this enzyme is secreted and that it may have an effector function in the host plant, suppressing H_2_O_2_-mediated defense signaling [44]. In CLas samples from Florida, homologs of the two prophage regions FP1 and FP2 have also been found to form a recombinant, incomplete prophage variant iFP3. The presence of iFP3 was correlated with blotchy mottle symptoms observed in the plants, suggesting that it could be involved in disease development [76]. Thus, the prophages found in CLso could also contribute to the genome plasticity and carry genes involved in the bacteria-host interactions.

One of the haplotype C specific genes (AYJ09_01490) was found to be homologous to the MIF4G domain of eIFiso4G that is involved in virus-plant interactions in different plant species [55,77]. Thus, the phage-encoded small protein found in CLso haplotype C might play a role in the interaction with the host plant. Another hypothetical protein (AYJ09_01545) shared over 50% amino acid identity with a phage-related hypothetical protein from a *Bartonella* species. *Bartonella*, which belongs to Alphaproteobacteria and is closely related to ‘*Ca*. Liberibacter’ species, is an intracellular parasite and pathogen of humans and animals, and transmitted by insects and ticks. Thus, it is possible that this hypothetical protein found in CLso haplotype C could be involved in the psyllid transmission.

Of the ortholog groups that are present in the haplotypes A and B, and absent from the haplotype C, some may encode proteins that would be disadvantageous for the interactions of the haplotype C with the carrot plant or carrot psyllids, e.g. by acting as avirulence factors. Alternatively, some of these ortholog groups could encode proteins that are involved in specific interactions with the solanaceous host plants or the psyllid *B*. *cockerelli*. In this respect, the 11 ortholog groups that are present in both ZC1 and NZ1 but not in FIN114 or FIN111, are worth closer examination. These ortholog groups were annotated as hypothetical proteins, except the ortholog group 1003 that encodes for a DNA repair enzyme 3-methyladenine DNA glycosylase. This enzyme has been well characterized in *E*. *coli*, and in general, it confers protection to DNA by limiting mutagenic and clastogenic events [78–80]. For now, the substrate range of the 3-methyladenine DNA glycosylase encoded by ZC1 and NZ1 has not been tested, but the absence of this gene in FIN114 suggests that haplotype C might have a reduced DNA repair capacity and higher mutation frequency than the haplotypes A and B. The two copies (CKC_RS00980 and CKC_RS05675) of the ortholog group 22 in ZC1 were recently recognized as potential effectors, and CKC_RS05675 was shown to have a higher expression level in the haplotype B than in the haplotype A, possibly modifying the interaction with the psyllid *B*. *cockerelli* [81]. The putative small secreted proteins encoded by ortholog group 986 could also be considered as candidate effectors. The proteins encoded by ortholog group 987 are homologous to a phage-related hypothetical protein of *Bartonella*. As *Bartonella* species are insect-transmitted animal pathogens, these proteins might contribute to the bacteria-insect interaction. The proteins encoded by ortholog group 1000 and containing a DUF1640 domain are likely to have a phage origin. A DUF1640 superfamily protein of bacteriophage AKFV33, a putative biocontrol agent for Shiga toxin-producing *Escherichia coli* O157:H7, encodes a tail fiber protein which may play a role in bacterial surface recognition and adhesion [82].

In this study, two genomic sequences of CLso haplotype C were assembled and analyzed, and discussed in relation to the obligate parasitic lifestyle of CLso. The comparative genome analysis including three different haplotypes of CLso may help to identify potential haplotype-specific effectors. Since haplotype C is transmitted by a different psyllid vector and has a different plant host range than the haplotypes A and B, the differences found in both the gene content and genome organization may explain some of the differences in the interactions with plants and psyllids. Besides the mining of novel gene candidates, the new haplotype C genome sequences are also useful for applied research and diagnostics.

## Supporting information

S1 Table Carrot psyllid (*Trioza apicalis*) samples used for sequencing. (DOCX)

S2 Table Primers used for ‘*Candidatus* Liberibacter solanacearum’ haplotype C genome gap closure and for sub-cloning of the rRNA operons and the prophage regions. (DOCX)

S3 Table RefSeq protein datasets used in the phylogenetic analysis. (DOCX)

S1 Fig Amino acid sequence homology of the hypothetical protein AYJ09_01490 to the MIF4G domain of eIF(iso)4G. Alignment of sequences corresponding to AYJ09_01490 were performed using Clustal X and the secondary structure was predicted using Jpred 4. S.lyc, S.tub, S.pen, O.gla represent sequences from *Solanum lycopersicum*, *Solanum tuberosum*, *Solanum pennellii*, *Oryza glaberrima*, respectively, and 'X1/X2' represent different isoforms of eIF4G. The amino acid residues highlighted with colors have high conservation (above 30%) of similarity regarding the hydrophobicity character. The secondary structure predictions, helix (red) or coil (black), are displayed on the bottom row.
(TIF)
