# Supplementary material for: Genomic sequence of 'Candidatus Liberibacter solanacearum' haplotype C and its comparison with haplotype A and B genomes
Source: PLoS One. 2017 Feb 3;12(2):e0171531. doi: 10.1371/journal.pone.0171531 (PMC5291501; doi:10.1371/journal.pone.0171531)
Supplement: S1 Table — (DOCX) [file pone.0171531.s001.docx]

S1 Table. Carrot psyllid (*Trioza apicalis*) samples used for sequencing.

| Sample | Origin | Time of capture | Population | Sequencing unit | Sequencing method |
| --- | --- | --- | --- | --- | --- |
| FIN114 | Forssa,  Finland | June 2012 | Field  sample | Macrogen,  South Korea;  Expression Analysis Ltd, USA | 1.Illumina Hiseq2000, WGA, 432bp PE library  2.PacBio RS SMRT cell |
| FIN111 | Forssa,  Finland | June 2012 | Field  sample | Macrogen,  South Korea | 1.Illumina HiSeq2000, WGA, 670bp PE library  2.Illumina HiSeq2000, WGA, 3kb MP library |
