# Supplementary material for: Genomic sequence of 'Candidatus Liberibacter solanacearum' haplotype C and its comparison with haplotype A and B genomes
Source: PLoS One. 2017 Feb 3;12(2):e0171531. doi: 10.1371/journal.pone.0171531 (PMC5291501; doi:10.1371/journal.pone.0171531)
Supplement: S2 Table — (DOCX) [file pone.0171531.s002.docx]

**S2 Table.** Primers used for ‘*Candidatus* Liberibacter solanacearum’ haplotype C genome gap closure and for sub-cloning of the rRNA operons and the prophage regions.

| **Primer** | **Nucleotide Sequence 5'-3'** |
| --- | --- |
| **Primers for 16-23-5s rRNA cloning** |  |
| 16S_A_F | GAACTGATGAAAACGGAAGGTG |
| 16S_A_R | TTTCTTTACGCCCTTGTACTCG |
| 16S_B_1F | GAGAGCAAGGGCATAAGAAGAA |
| 16S_B_1R | TGAGAAACATTCTGCTCTTCCA |
| 16S_B_2F | GCAAGGGCATAAGAAGAAATTG |
| 16S_B_2R | CCAAATCATCAAGGAAACCAAA |
| 16S_B_F | GTGTTACCCGCTAGATATCCG |
| 16S_B_R | ATAGCGGATTGTACCATTGGAC |
| 16S_C_1F | CGTGGAAAACCTCTAAATCCAG |
| 16S_C_1R | TCGCATAGAAAAAGGGTTAGGA |
| 16S_C_2F | ACGGATATTCGTGCTTTTGTTT |
| 16S_C_2R | CGAAGAGAAACCTTGCGTCTAT |
| 16S_C_F | TTTACGTCATTCTTTTGCCACT |
| 16S_C_R | CTCCAGCAAAGAATGTTGTGT |
| **Primers for gap closure** |  |
| 1L_59F21 | AATAGGATATTCGCCAAGTGT |
| 1L_779R22 | TAATAGGATATTCGCCAAGTGT |
| 1R_135F21 | TGGCACTAACAGTAGATGGAA |
| 1R_298F22 | TCAACCAATAAACGGAATCACC |
| 2L_723R22 | ACATGTCTACTAGAGAAGGCTA |
| 2R_257F21 | ACATCATTCGTAGTTGGCAAT |
| 3L_635R20 | GGTTATGGACTTTGGTGCTT |
| 3R_110F20 | TCTTGTTATCCCGACAGCCA |
| 3R_39F22 | AGGATATAATCTGCGATACACA |
| 3R_79F21 | CACATGCGTTATTAAATGGAT |
| 4L_1057R21 | TAATTGCAGGTGAAAGACGTT |
| 4L_1356R21 | TGAAAGACGTTTTCGAGCAGC |
| 4L_677R23 | CGTGCTTTAATTTTAACTTCTGA |
| 4R_236F22 | CAGAAAATAAAATTGCAGCATC |
| 5L_622R22 | CGTGATAAGCTAGAAGACGCTA |
| 5R_126F22 | ATTACGAAGGTAGGAGGCGTTT |
| 5R_994F23 | TGAGTAGAGATGATTTTCGCTTG |
| 6L_2158R23 | CCCCTAACTTAATTACCCGCCTA |
| 6L_658R23 | CTATACTTCAAAGCCTACCACCT |
| 6R_175F21 | CCAGAACCGCCTTAATGGTGA |
| 6R_195F24 | GCGATTTATTATATGCTATAGGTG |
| 6R_976F22 | TCATCCTGAACGATATACCGCA |
| 7L_1028R22 | GGAGATTCTGGAAACAACGGAT |
| 7L_1429R22 | GTAACCGCAGCATTACGAAAAC |
| 7L_678R19 | GATTCTGGAAACAACGGAT |
| 7R_135F22 | TATGCCCCATCCTATTTCACCT |
| 8L_624R22 | TTGATGCCCTAGAAGATGGAAC |
| 8R_121F22 | CTTTAATGCCATATCCGACACC |
| 9L_684R22 | TATTCTAATCCCATCTCCCCTT |
| 9R_139F21 | ATCTTTGCTTGTCTATGGCTT |
| 9R_1631R22 | TATCCTCTGTTCGTCCTGTTGC |
| 9R_1654R22 | AACGGCATCTAATACTATTGCT |
| 9R_1934R21 | TCCACGATGAGAATCCACCAC |
| 9R_27F22 | CGTAAGGATAAGAGGCAACGAA |
| 9R_283F22 | TTTGATAAAAGTAGGGCGTCCA |
| 9R_314F22 | GAGTAGCGCGAAAATATTTGGG |
| 9R_359F22 | CTATGCGTTTTGAAATATCACC |
| 9R_49F22 | TGTTGATATGGAATTCGTGCTT |
| 9R_779F22 | AATTTCTATAGCGGTAACACCA |
| 9R_829R22 | AAACTACGGAAGATCAATGGTG |
| 10L_1147R20 | TTAATCGCCATAGCCGCAAA |
| 10L_2497R22 | TATATTACCGCATCAAAAGCAA |
| 10L_766R22 | CCAAAATAACCGCCTATACAGA |
| 10R_1316R20 | TGGTATCATCCAAAGCGTTC |
| 10R_217F22 | TTTAGTTGTTGATAGCGAGCAT |
| 10R_69F20 | TGGTATCATCCAAAGCGTTC |
| 11L_1313R22 | TAATAACCTGCTTGAACCCCTC |
| 11L_426R22 | GCACCCTTTACATATAAACTCG |
| 11L_602R22 | TACTTTTATGAGCTACCGCAAA |
| 11L_885R21 | TATATCCTTGAGCTTCGTCCC |
| 11R_186F21 | ATAATGGCGAATACATAGGAA |
| 12L_611R22 | AGTATATTTTGTAGCGTAACCA |
| 12R_110F22 | TGGTAATGCGAGTTCCATCGTT |
| 12R_256F22 | CAGGCTTAACACATGCAAGTCG |
| 13L_1514R22 | CGAATTAAACCACATGCTCCAC |
| 13L_667R22 | GGTAAGGTTCTTCGTGTTGCAT |
| 13R_248F22 | CTAGTACCGTGAGGGAAAGGTG |
| 14L_566R22 | GGTTAGGATCAGTAAGGCGGTA |
| 14R_303F22 | TGAACAGTAATCTATCACGTCT |
| 15L_639R22 | AAACTTTATCATTGTCACCGTA |
| 15R_255F21 | GATATGCAGGTTTAGCAGTGG |
| 16L_595R21 | AGAGCCAATAAGAATCACGAA |
| 16R_300F22 | TCCGCATTATGTAGAAAAGCTC |
| 16R_449F22 | AGCGTTGTTGATAATTAAGGGA |
| 17L_1861R22 | ATTATGGAAGGTACAAGGCAGA |
| 17L_509R19 | GGCCATAACCAATAGTCCA |
| 17R_137F22 | TATTTCCCATCCGATTTCACCT |
| 18L_521R21 | AGGATGTAATATTGACGCTCT |
| 18R_76F22 | AAATTACAGATAACACCGCATC |
| 1920_3KF | TTTCAGCCTGATGTTTTACGTT |
| 1920_3KR | GTTTTAGGCTTAACCTCACCAG |
| 1920_5KF | GTTCTGCTAAATATAGGCGTCA |
| 1920_5KR | CTTCAAAACTATTGAGGGGTGT |
| 1920_7KF | TTAGGTGTTGTGCTATGGGTT |
| 1920_7KR | CGAAAGGCATTAGAGTAAGAGC |
| 1920_8KF | TTTTCAAGATGCGATACGTGAC |
| 1920_8KR | TTCCATTCTTTTCTGCTCGTCT |
| 19L_631R22 | CGCTTCTTTATTAACATCTCCC |
| 19R_239F22 | TTTCAGCCTGATGTTTTACGTT |
| 19R_298F22 | AAAGCTATCATTGATGCCGAAC |
| 19R_298F22 | AAAGCTATCATTGATGCCGAAC |
| 20L_570R21 | TAGCACCGTAAAATCAAGAGC |
| 20R_65F22 | GAGACATTCTATTATAGGCAAC |
| 21L_500R21 | CACGATAATAATTACCCGACT |
| 21R_178F19 | CAAACGCCTCAATCAACGA |
| 22L_580R22 | TTGGGAATTATTGCTTGTGCTT |
| 22R_166F21 | CATTATATCGGGGAAAGAAGC |
| 23L_13F21 | CATTGTACATGATACGCAGGA |
| 23L_2667R22 | TTGAAGACGCTTAAGATCGCTA |
| 23L_474F22 | TGTTAAGTATGGTCGATATTGC |
| 23L_669R21 | TCGCAACTGTTAAGTATGGTC |
| 23R_304F22 | AAAGCTCCTGAAATAAAACTGG |
| 24L_587R20 | CCTCAGATGCACATATACCG |
| 24R_72F22 | TATTTCTCTGCAGCTCTCACCT |
| 26L_607R20 | TTAAATGGTTCTTTGCCTCC |
| 26R_105F22 | AAGCCACTAAAATGATAAACGA |
| 27L_591R22 | TACTAAGGGGCATTATAGCAAC |
| 27R_120F22 | CCTTCCATTTTATATTGGCAAG |
| 27R_647F21 | TTACACGTCATAGTTCGCCTT |
| 28L_1716R22 | AAAATTCTCTGGCGATAAGCTC |
| 28L_550R22 | TGTCTTTTATTTCGGTCTCTGC |
| 28R_123F22 | TTTACGAGGAGCAAAGATTGGG |
| 28R_77F22 | TAAGTAAACAACATTGGAGCAG |
| 29_int_1F | TTCGGAAAGATAAGACACAAGC |
| 29_int_1R | GCTTGTGTCTTATCTTTCCGAA |
| 29_int_2F | GAAGGCATGAATTGTTGGTGT |
| 29_int_2R | GAGAACGCAATTTGATTACCAG |
| 29_int_3F | CTCTGGTAATCAAATTGCGTTC |
| 29_int_3R | TTTCCCCTATAAACATTCCGAT |
| 29_int_4F | GTCTTAAGCTCTTCTGCAGCAA |
| 29_int_4R | CAAAAGAGAGCCAACATACGAT |
| 29_int_5F | TTATCACAGCCTCTACAAACCC |
| 29_int_5R | AATGACTACTACGCCAAGACCA |
| 29_int_6F | AATTGTTATTCATGCCCGTCT |
| 29L_769R22 | GAGAACGCAATTTGATTACCAG |
| 29R_108F22 | AAGATATGGAGCTTATGACGTT |
| 30L_207R20 | TAATCGCATTAAACCTCTCG |
| 30L_666R21 | GAAATCGTTATTCTAGCCCAT |
| 30R_90F21 | ATACATAATATGAGACCTGCT |
| 31L_537R19 | GGTATAAGCGAAAAGCCAT |
| 31R_211F21 | GACCTTAAAATCATAGGGCAA |
| 32L_545R20 | CCTCTTTAATCATTCGCCTT |
| 32R_201F22 | GGGCGTTGATTCTACTATGCTT |
| 32R_445F22 | GGGAATAGTAGCGATAACCTGT |
| 33L_1003R22 | TTACAAGGCACTATCATCGAGA |
| 33L_731R22 | AGTTGTCGCCTAGATAAACCAC |
| 33R_176F22 | TTGTAAAGTGTTATGCAGCAAG |
| 34L_647R21 | CGCAAGATTAATGGTAACTCG |
| 34R_137F19 | GGAATAGATGGCCGTGCTC |
| 35L_685R22 | GAATAATATGTTTTCCGGTGGTGG |
| 35R_278F21 | CGACGCCTTAACTAATCTCCA |
| 35R_88F21 | TCTTAATGCTATCCTCGGCTT |
| 36L_1030R21 | GATCGTTGCACATTTAACACC |
| 36L_680R21 | GATCGTTGCACATTTAACACC |
| 36R_149F21 | CACTAGGCAAAACAATATCCG |
| 37L_694R22 | TTTCCCATCAAATTATCGGTCA |
| 37R_118F22 | AAAATTCGACCAGAAACACACC |
| 38L_595R22 | TCCATGTTTTACCTCTCCCGAT |
| 38L_188F22 | ACTTTATATACGGGCATAACGA |
| 38L_189F20 | CACAAAGGACGGAATACACT |
| 39L_1432R22 | ATCTAGTATATCGCATTTCCCT |
| 39L_627R20 | TAATTATTGGAGCAGCGAGT |
| 39R_427F21 | AAAATTGCCATGTACTTCTGC |
| 39R_89F22 | TTATCAACAGTCATAGGCGAGA |
| 40L_1416R23 | CGCAAGAATATAATGACATGCTC |
| 40L_603R21 | TTTGTTTTCATTGCCTCTGCT |
| 40R_124F19 | GAATATTTTCCGAACCCAT |
| 41L_556R18 | AATTGATACGGCAGTGGT |
| 41R_231F21 | TAAATCCGTAGCATGTCGAAC |
| 42L_718R22 | CTTTAATTTCTGAAAAGCCTGT |
| 42R_221F22 | GCAATAGCCAAAGTTAATCCCT |
| 42R_89F22 | TCCCGTCATTGGATTTATGCTT |
| 43L_1120R22 | GTGTATTAGATGCGGCTCCTGT |
| 43L_131R22 | ATTTCGTGTCTATGGAACTCCT |
| 43L_622R21 | GAATCGAACCCCTCTTTCCAG |
| 43L_802R22 | ATATCAGATGATCCGCAAGAGC |
| 43R_14F22 | ACTAATAGCAGAAACGACGAAC |
| 44L_573R22 | ACTGCTCTTCATTAATACCCTT |
| 44R_71F22 | ACTAAATAATCCAGCAGAACCC |
| 45L_648R22 | CAACTTCTGCCTTTCTTCTTGC |
| 45R_1562R22 | ATCCTCTTTCACATTCCGCTTG |
| 45R_86F23 | ATCTGATAGCCAAATACTGCATG |
| 46L_1277R22 | GATCTTCACGTCTTTCACCGAT |
| 46L_705R21 | CGTATACCAATTCCGCCACGA |
| 46R_135F22 | TGGATTTTAGATTTCGGACCTG |
| 46R_312F22 | ATTGTTTCATTCCCATAAAGCG |
| 47L_1440R23 | GTGCATAAAATGGATCTATGTGG |
| 47L_717R22 | GTTCATCCTATCATTGTGCCAT |
| 47R_203F20 | CATTGGCGTTAACAGACCAG |
| 48L_755R22 | CAAGGCTCACAATAAGATCGTT |
| 48R_71F22 | AACTTCTATGAAAATAGCCGAT |
| LDP_1_R | GAGCCGCATCAATCCTATGGAG |
| LDP_2_L | TCCACCTTGTTCAGCGGTCA |
| LDP_2L | GATCAGGAACAGATATCCGAGA |
| LDP_5_R | TAGACGTGATGAAGTGCGGGAA |
| LDP_6_L | CAATCGGAGCTATGTCAGTTGG |
| LDP_12_R | TCTCCCTTGTGCGGAATACAGG |
| LDP_13_L | GCGGATTGTACCATTGGACCAC |
| LDP_19R | AAAGCTATCATTGATGCCGAA |
| LDP_20L | ATGTTTGTAGGACGATTAGGTG |
| LDP_22R | AGCCGATGATTTCGTATACACA |
| LDP_23L | TATCTTTTAGTTGCGTCTCTGG |
| LDP_24_R | TCGTCAATTATGCCCTCCAGT |
| LDP_26_L | ACCGTCTGATCAAGATATGCAA |
| LDP_28_R | TGTTGATGAAGGCATGACGAGA |
| LDP_30_L | GCGGATGAGGTTAATGTAGGGA |
| LDP_32_R | CTGGAACAAGCCATTTTCGTCT |
| LDP_33_L | GAGAATCGTTGTGGCTGTTCG |
| LDP_48R1 | ATCGGCAATTTCAATAAACTGG |
| LDP_48R2 | TTTTCGTGTTTACCGGCAT |
| 114_1L | ATCACTGGCTATTGGAAATGCT |
| 114_2L | AAGCGTGATACCAATATGAGGG |
| 114_2R | AACATGCTGACCAATGAGATGA |
| 114_3L | TTGCGGGTATAATGAAGGTTGT |
| 114_3R | AAATTCACCTTACGACCCTCTG |
| 114_4L | ATCTGAGATTGAGTGTTGTGGG |
| 114_4R | TAAACACTTGCCAGAAATCACC |
| 114_5L | GGTGACAGGTGAAAGTATGACG |
| 114_5R | AACAGCAGCTAATCCAATACCA |
| 114_5L2 | ACAGTGAGAAAAGCATACGGAA |
| 114_5L3 | TCAATTCTTTCGCTCAGACAGT |
| 114_6L | GCTATTTCTAATCCGAGTCGCT |
| 114_6R2 | CATGACTAACGGTGAAAGCAAG |
| 114_6R3 | AAGCCACAGAAGAAAGAACCAC |
| 114_7L | ATTTCTTTGTGCTCTTCCAGGT |
| 114_7R | GCGCTCCTAACAAACATCATAA |
| 114_8L | TCTCCTATATGCCCGAACAAAC |
| 114_8R | ATCAAGGAAACGACGTCTTTCT |
| 114_11R | GACCGTCTCTTCCTCCTATTTC |
| 114_12L | ACCACCAATCTACAAACATCGT |
| 114_12R | GTGGTGGATTGGTGATGACTAA |
| 114_13L | GACAACAACACGACTTATTCCG |
| 114_14L | ACAACTACAGAACGTATCGGGA |
| 114_14R | TCACGACAAGGTAATGCAACTT |
| 114_2R_1 | ACCCTTTACAAGAGAGGATTGC |
| 114_3L_1 | TGAAAGGCGGGTATTAGGTTTT |
| 114_antigen | TGCTAAGCGTCGTATCAAGAAT |
| 114_15R | TATGGAGGATCAGTAGGTGCTT |
| 114_PA3_14F | TTGTCTTCTGATAACGCCTTGT |
| 114_PA3_14R | ATCTACAATCATACGGGGCAAG |
| 114_PA3_2F | TGGTGAGCTTGAGAAAGGATTA |
| 114_PA3_2R | TAATCCTTTCTCAAGCTCACCA |
| 17R18L_PW1 | ACCGCTTCTCCTTCCATTTTAT |
| 17R18L_PW2 | AAGCATAAGGAAATGTGTTGCA |
| 2R3L_PW1 | CTATGCTCCAGATAGTCGTCAC |
| 5R6L_PW1 | GGGTTGTTAGGTTGTGTCAAAA |
| 5R6L_PW2 | ATGGTAAAATTGTTCTTGGGCC |
| 5R6L_PW3 | AATCAACTGGTTCTTGTCGAGT |
| 10RLPS_PW1 | ATTAGCGGAAAGTATAGGTGCG |
| 10RLPS_PW2 | CCACTAAACTAATGGGGTCAGG |
| 10RLPS_PW3 | TGGTGCTAATAGTGCGATTGAT |
| 10RLPS_PW4 | GACAGAAGTATGGTGGTATCCC |
| 10RLPS_PW5 | TGTTATGCCTGGTGTTCTCATT |
| 14Rpo_PW1 | CCTTGAAGCGTGTGATACAAAA |
| 14Rpo_PW2 | CTTCACCACCATTATCAGCTCT |
| 13R14L_PW1 | GCTGAAGTTGTATTGCCTCATG |
| 13R14L_PW2 | TAACTTAAGACGCCCTAACCCC |
| 13R14L_PW3 | GAGAGGAAAGAACAGCCGATTA |
| 13R14L_PW4 | CGTATCTTTAGGTGGCTGTGAT |
| 13R14L_PW5 | ACTGTATTCGAGTGGCTTTTGA |
| 13R14L_PW6 | TAAGAGATGTAACGACGAGTGC |
| 13R14L_PW7 | GGACGCTGGCTTTTATAGGTAT |
| 13R14L_PW8 | TTCCCAGAGATAAGAGGAACGA |
| 13R14L_PW9 | TCTCCTTAACGGTCTGTATCCA |
| 13R14L_PW10 | GGAGATTCATGCACAGACGTTA |
| 8RLPS_PW1 | GGATTGAAAGACTGTCCACTGA |
| 8RLPS_PW2 | TTTTACCTGCTCCATTATCCCC |
| 8RLPS_PW3 | CCAGTTGGTTCAGTATCGCATT |
| 8RLPS_PW4 | GTGGCATTGTTCCCTCTGTC |
| 8RLPS_PW5 | TATGCTGGTTCGTTTATGGTGT |
| 8RLPS_PW6 | TCATTAGGGTAGGGAAGACGAG |
| 8RLPS_PW7 | TAAAGATTGCGTTCTGGAAGGT |
| 8RLPS_PW8 | TCAGCTTGCGATACTGACTTAG |
| 8RLPS_PW9 | ATGAAACGGCTGATTACCCTAG |
| 8RLPS_PW10 | ACCAGTCGCCATATTAAACAGC |
| 8RLPS_PW11 | ACAGTTCGTTATTGTCGGATGT |
| 8RLPS_PW12 | ACGTCTTGTCAACCGAATATGA |
| 8RLPS_PW13 | ACATCCGACAATAACGAACTGT |
| 9R_PW1 | TGGGGATATAGCCTTATGTGCA |
| 9R_PW2 | TGTATGTACCACGATCAAGCTC |
| **Primers for prophage regions** |  |
| pha_1F | ACACTTGAAACAAATCAAGCAG |
| pha_2F | CCTCCTCATAAAGAAAGGCTTG |
| pha_3F | GTGCGAAAGAAATCATCACCA |
| pha_4F | CTAATTGTTCTTTTGTCGCCAT |
| pha_5F | TTACCGTCTTTATCAACGCTCA |
| pha_6F | ACATCGTATCATCCGTTATTGC |
| pha_7F | AGATAAAATCTTGACCCATCGC |
| pha_8F | CTCAGTGCCTTAACTTTATCCG |
| pha_9F | TTTCAGCCACAGAATTTACCAC |
| pha_10F | TATACCATCGTTCTCACCGTTC |
| pha_11F | AAGCCTTAAATCAACACGCTTC |
| pha_12F | TACATAACGCCAAGATTGAAGC |
| pha_13F | ATTATCTCATCGCTGAAAACACC |
| pha_14F | CGCATACGATTACCTAACGTCT |
| pha_15F | TTTAAAAGCCATTTCCCCAGT |
| pha_16F | CGCTTTTATTGTATGAGGTGGT |
| pha_17F | TATAACGGTTGGTTTTGTCCCT |
| pha_18F | TTACAACGGAGAAAGGAGCAAC |
| pha_19F | ATTCAACCCCGTATCAGAGC |
| pha_20F | GTATCCAAGCACTAATTCGGTT |
| pha_21F | AAATCAACGGATGAACTTTTGC |
| pha_22F | ACTCTTTTGGATTCAAGGGCTA |
| pha_23F | AAATGCAGTGATATAGCCGAAC |
| pha_24F | CCCTTATTCAAATACCAGCAAG |
| pha_25F | AATCTCTGCCAGTCATTAAGGG |
| pha_26F | CTTCATCGTACGTTGATCCTCG |
| pha_27F | GACTTTTGGGTGTCTGCTTCTT |
| pha_28F | TGGGATTCAAAGGGATAGAGGA |
| pha_29F | CTGCTTTTCTTAATCCGTGAGC |
| pha_30F | AGCTCTACATTTATCGGGAAGC |
| pha_31F | ATTCCATGCATCACAAGACTCA |
| pha_32F | CCCATATACGATCTAGCCTTGC |
| pha_33F | CCCATCATTCGCTCAATAGACT |
| pha_34F | GAGCAAACCTAAATATGCCACC |
| pha_35F | AGATAGGTGTAAAGAAGCGGGA |
| pha_36F | GTGTCGTCAATCACCTTTTCCT |
| pha_1R | TAGGGCATCACAACACTTACGG |
| pha_2R | GGAAGATACCGAGTGAGAGCAG |
| pha_3R | CAATTCGGTAATCCTCTTCCCT |
| pha_4R | ATTTAAGCAATCAGAAGCCCAT |
| pha_5R | CCACAAATTACTTTAACGGCAT |
| pha_6R | TTTATCGGTGCATTACTGAGGG |
| pha_7R | ATGTTGATTATCGCAAGTCGTT |
| pha_8R | CAAGGGTGAACTTTTAGGCTGT |
| pha_9R | AATTAAAGGAGAGCAAGCGAAC |
| pha_10R | CCCCAAGTATAGGAGGTAGCAA |
| pha_11R | TAAATCTCGTTATGGGGAGCAG |
| pha_12R | TGGCAGAGAAGCATTTATGG |
| pha_13R | GTACAAATCCAACGGTGAGG |
| pha_14R | CTAATTCCATGACTTTGCCGAT |
| pha_15R | TTGTAGCGTTTATCATCCCAGT |
| pha_16R | TGTCTGCGTGTTATTTTAGCAA |
| pha_17R | GGCATACCCCGATTACTGG |
| pha_18R | GCGATAAAAGACCATTGAGAGC |
| pha_19R | TATATGGGTGAGCAAGACGGTA |
| pha_20R | CACCCGTTAGATATTCAGTTGC |
| pha_21R | GAAAACAACCGAATTAGTGCTT |
| pha_22R | ATGCACAATAAACACTCGCTCA |
| pha_23R | TTAGGAAAGCCATTAGACTGGA |
| pha_24R | CTTTATCGTCTCGTATGCGTTC |
| pha_25R | GAATCTTCTTGTTGGCTTCAGG |
| pha_26R | CGCACACTAAATGATATTTTGGAC |
| pha_27R | TCTACAATCATACGGGGCAAGA |
| pha_28R | GAGAAGTCGACTGAGGGAGAG |
| pha_29R | GCCAATCTACGCTTTTGATGTT |
| pha_30R | AAGCGGTTGAAGATGACACTAA |
| pha_31R | ACACCCTCCTTAGTCATCTCAT |
| pha_32R | AGATGCTGTAACGCTGTCTTTC |
| pha_33R | CTGATTGAGTTGTTCCTCCCTT |
| pha_34R | GGTTTTCTTGCTCTTCGGTTTT |
| pha_35R | TCGTAACAGAGGTCAGGTGAAA |
| pha_36R | AACGCATCCCCTGTATCAATAG |
| pha_37R | GCGAAGTCTTTCTGTTCTCTCC |
| pha_38R | CTATTGATACAGGGGATGCGTT |
| pha_39R | AAAAGCCAAAAGTTTCAAAGCC |
| pha_40R | ATCAGAAGTCCACGCAACTAAA |
| pha_41R | ACAGGCTTAATCTTGTCGTTCA |
| PA5F_PA3_PW1 | TGTTCTCATCCCATCTGAAGTT |
| PA5F_PA3_PW2 | AGCCCTTGATATTCAGCATTCT |
| PA40RPA3_PW1 | AACACTAGGAAAAGACGCCATG |
| PA40RPA3_PW2 | GTCTTCAGCAGGGGATACTTTT |
| PA40RPA3_PW3 | CAACCTAACAAAAGATGAGGCG |
| PA40RPA3_PW4 | GTTTAAGCCGTCAGATGCTCAT |
| PA40RPA3_PW5 | GTAACCGCTACAGATATGGCTT |
| PA40RPA3_PW6 | CAAATCACTGTTCTCGTACCCT |
| PA40RPA3_PW7 | CAACCACTACCCATGTAACACT |
| PA26F14R_PW1 | TTTAAAACTATGGGGCGGAGTT |
| PA26F14R_PW2 | CCATGCAGAACTTCAAAAGCAT |
| PA26F14R_PW3 | TAGTCATCGGATTAAAGGGCAC |
| PA26F14R_PW4 | CGATAATGCAAGAACGAACACA |
| PA26F14R_PW5 | CGATAATGCAAGAACGAACACA |
| PA26F14R_PW6 | GATGTTCTCCTACGCCACAGAC |
| PA26F14R_PW7 | CTTTTATCACCGTGGCGTAAAG |
| PA26F14R_PW8 | TCAGTTAAACCTCCACTACAGC |
| PA26F14R_PW9 | AGAAACTTTCCCTATACACCGC |
| phaB_ex_PW1 | TACTGTTGATGTTGCTCGAGTT |
| phaB_ex_PW2 | CACAGGGTTGAGAAGTTCTGAT |
| phaB_ex_PW3 | AGCAAGCAAATATAACGCCATG |
| phaB_ex_PW4 | CTACGACAGTCCACATTCAACC |
| phaB_ex_PW5 | GAGCCTTTGTAGCCTCTATGAG |
| phaB_ex_PW6 | TATCCCTTGTTGTTGCTTACGT |
| 15R_PW1 | CTGCTTGTGATACAGTCTTCCA |
| 15R_PW2 | ACGGAATCCTCAAACAAGACAT |
| 5R5L2_PW1 | TACTGTTGATGTTGCTCGAGTT |
| 5R5L2_PW2 | ACTCGGCATTGTTCTTGAAAAC |
| 5R5L2_PW3 | GTTTGTGAGAGGAGTGAACGTA |
| 5R5L2_PW4 | GAACTAGCACGGATAGAGGGTA |
| 6L6R2_PW1 | CCTCCGCATATTCTAAAGCAGA |
| 6L6R2_PW2 | GTTTTGGGGTTGTTAGGTTGTG |
| 25_1F | TCGATAAGATCCGATTGGTCA |
| 25_1R | AGATGCTTCTTTGAAAATAGCGTA |
| 25_2F | TCCGTTTTCTATTTTACTTATCCCT |
| 25_2R | ATCTTCAATCGGTTATGTCCAC |
| 25L_611R22 | TGATAGAGCGTATGAAGACGTT |
| 25R_54F22 | TTGATTCAGTAGAAATACCGAT |
