# Supplementary material for: Genomic sequence of 'Candidatus Liberibacter solanacearum' haplotype C and its comparison with haplotype A and B genomes
Source: PLoS One. 2017 Feb 3;12(2):e0171531. doi: 10.1371/journal.pone.0171531 (PMC5291501; doi:10.1371/journal.pone.0171531)
Supplement: S3 Table — (DOCX) [file pone.0171531.s003.docx]

**S3 Table.** RefSeq protein datasets used in the phylogenetic analysis.

| Bacterial strain | RefSeq accession number |
| --- | --- |
| *Candidatus* Liberibacter africanus str. PTSAPSY | GCF_001021085.1 |
| *Candidatus* Liberibacter americanus str. Sao Paulo  *Candidatus* Liberibacter americanus str. PW_SP | GCF_000496595.1  GCF_000350385.1 |
| *Candidatus* Liberibacter asiaticus str. gxpsy | GCF_000346595.1 |
| *Candidatus* Liberibacter asiaticus str. psy62 | GCF_000023765.2 |
| *Candidatus* Liberibacter asiaticus str. A4 | GCF_000590865.2 |
| *Candidatus* Liberibacter asiaticus str. Ishi-1  *Candidatus* Liberibacter asiaticus str. FL17  *Candidatus* Liberibacter asiaticus str. HHCA  *Candidatus* Liberibacter asiaticus str. YCPsy  *Candidatus* Liberibacter asiaticus str. SGCA5 | GCF_000829355.1  GCF_000820625.1  GCF_000724755.1  GCF_001296945.1  GCF_001430705.1 |
| *Candidatus* Liberibacter solanacearum str. ZC1 | GCF_000183665.1 |
| *Candidatus* Liberibacter solanacearum str. NZ1 | GCF_000968085.1 |
| *Candidatus* Liberibacter solanacearum str. HenneA | GCF_000968075.1 |
| *Candidatus* Liberibacter solanacearum str. RSTM  *Candidatus* Liberibacter solanacearum str. R1 | GCF_001414235.1  GCF_000756225.1 |
| *Liberibacter crescens* str. BT0 | GCF_001543305.1 |
| *Liberibacter crescens* str. BT1 | GCF_000325745.1 |
| *Agrobacterium tumefaciens* str. Ach5 | GCF_000971565.1 |
| *Sinorhizobium meliloti* str. 1021  *Brucella melitensis* biovar Abortus 2308  *Brucella abortus* str. S19  *Bartonella quintana* str. RM-11  *Bartonella bacilliformis* str. KC583  *Bartonella henselae* str. Houston-1  *Bartonella vinsonii* subsp. berkhoffii str. Winnie  *Mesorhizobium loti* str. MAFF303099  *Mesorhizobium opportunistum* str. WSM2075  *Phyllobacterium* sp. str. UNC302MFCol5.2  *Phyllobacterium* sp. str. YR531 | GCF_000006965.1  GCF_000054005.1  GCF_000018725.1  GCF_000294715.1  GCF_000015445.1  GCF_000046705.1  GCF_000341385.1  GCF_000009625.1  GCF_000176035.2  GCF_000686005.1  GCF_000282595.1 |
| *Rhodospirillum rubrum* str. ATCC 11170 | GCF_000013085.1 |
