# Supplementary figures and images for: Genomic sequence of 'Candidatus Liberibacter solanacearum' haplotype C and its comparison with haplotype A and B genomes
Source: PLoS One. 2017 Feb 3;12(2):e0171531. doi: 10.1371/journal.pone.0171531 (PMC5291501; doi:10.1371/journal.pone.0171531)

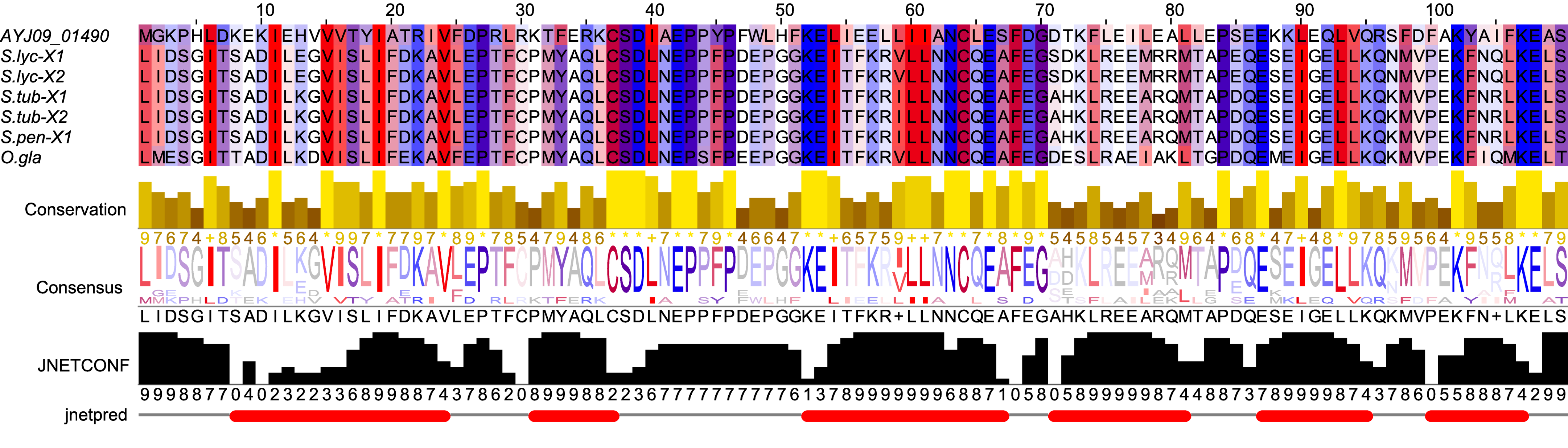

Supplement: S1 Fig — Alignment of sequences corresponding to AYJ09_01490 were performed using Clustal X and the secondary structure was predicted using Jpred 4. S.lyc, S.tub, S.pen, O.gla represent sequences from Solanum lycopersicum, Solanum tuberosum, Solanum pennellii, Oryza glaberrima, respectively, and 'X1/X2' represent different isoforms of eIF4G. The amino acid residues highlighted with colors have high conservation (above 30%) of similarity regarding the hydrophobicity character. The secondary structure predictions, helix (red) or coil (black), are displayed on the bottom row. (TIF) [file pone.0171531.s004.tif]
